# Improving the retention rate for residential treatment of substance abuse by sequential intervention for social anxiety

**DOI:** 10.1186/1471-244X-14-43

**Published:** 2014-02-17

**Authors:** Petra K Staiger, Michael Kyrios, James S Williams, Nicolas Kambouropoulos, Alexandra Howard, Stefan Gruenert

**Affiliations:** 1 Deakin University, School of Psychology, Faculty of Health, Medicine, Nursing and Behavioural Sciences, Burwood, Australia; 2 Swinburne University, Brain and Psychological Sciences Research Centre, Hawthorn, Australia; 3 Odyssey House, Victoria, Richmond, Australia

**Keywords:** Substance dependence, Social anxiety, Treatment retention, Residential drug treatment, Therapeutic community, Randomised control trial

## Abstract

**Background:**

Residential drug rehabilitation is often seen as a treatment of last resort for people with severe substance abuse issues. These clients present with more severe symptoms, and frequent psychiatric comorbidities relative to outpatients. Given the complex nature of this client group, a high proportion of clients seeking treatment often do not enter treatment, and of those who do, many exit prematurely. Given the highly social nature of residential drug rehabilitation services, it has been argued that social anxieties might decrease the likelihood of an individual entering treatment, or increase the likelihood of them prematurely exiting treatment. The current paper reports on the protocol of a Randomised Control Trial which examined whether treatment of social anxiety prior to entry to treatment improves entry rates and retention in residential drug rehabilitation.

**Method/design:**

A Randomised Control Trial comparing a social skills treatment with a treatment as usual control group was employed. The social skills training program was based on the principles of Cognitive Behaviour Therapy, and was adapted from Ron Rapee’s social skills training program. A permutated block randomisation procedure was utilised. Participants are followed up at the completion of the program (or baseline plus six weeks for controls) and at three months following entry into residential rehabilitation (or six months post-baseline for participants who do not enter treatment).

**Discussion:**

The current study could potentially have implications for addressing social anxiety within residential drug treatment services in order to improve entry and retention in treatment. The results might suggest that the use of additional screening tools in intake assessments, a focus on coping with social anxieties in support groups for clients waiting to enter treatment, and greater awareness of social anxiety issues is warranted.

**Australian New Zealand clinical trials registry:**

Australian New Zealand Clinical Trials Registry (ACTRN) registration number: ACTRN12611000579998

## Background

Treatment engagement and retention in residential drug rehabilitation settings for individuals with substance misuse and psychiatric comorbidities is variable [1-4]. Studies have implicated mental health conditions such as social anxiety as having a negative impact on engagement and retention [5]. As residential drug rehabilitation is often the last resort for people with severe substance misuse problems, effective management of negative prognostic factors would be a significant contribution to improved treatment outcomes. In Australia, as elsewhere, residential drug treatment is generally provided by Therapeutic Communities (TC). TCs are intense psychosocial based interventions which are grounded in the assumption that substance misuse is a dysfunction of lifestyle and character development involving the whole person, resulting in a range of inter and intra personal difficulties, which are most effectively addressed in long term treatment in a community comprised of therapists and peers (i.e., other uses) [6,7]. Treatment in TCs involves clients moving through a number of treatment stages over a six to 15 month period. Each stage is associated with specific learning objectives, which facilitate transition to subsequent stages.

Individuals attending residential drug treatment generally present with increased severity of symptoms e.g., [8] and incidence of psychiatric co-morbidity e.g., [9] relative to outpatients, and are more likely to have significant social problems [10]. Unsurprisingly, individuals with co-occurring psychiatric disorders are at greater risk of poor treatment retention e.g., [11,12] than those without such comorbidities. Thus, given the complex nature of patients attending TCs, treatment dropout is high. For example, Polimeni, Moore and Gruenert [13] examined dropout rates from an Australian TC over a 10 year period and found that approximately 45% of patients dropped out of treatment in the first five weeks. Australian Institute of Health and Workforce Studies revealed that four percent of patients stay in residential treatment less than one day, and 40% less than one month [14]. This low rate of retention is mirrored in data collected in other Western countries [15,16].

Although the positive treatment efficacy of TCs has been demonstrated in a number of studies [17,18], the high drop-out rate of patients in the first few weeks of treatment is of particular concern, especially in light of a substantial body of evidence which has found that for residential treatments, client retention of at least three months is associated with improved outcomes [16,19-22]. It has been suggested that treatment factors affecting drop-out might differ between treatment modalities. In an analysis of predictors of client retention in TCs, Condelli and De Leon [23] found that clients who spent a significant amount of time with large groups of people, rather than alone prior to drug use were more likely to stay longer in treatment. Similarly, in a retrospective review, Johns, Baker, Webster and Lewin [15] found that clients who stayed in treatment less than 90 days were more likely to have reported spending their free time alone, or with one other person than clients who stayed in treatment longer than 90 days. It is unsurprising given the social nature of the TCs, that clients who have previously experienced difficulties interacting with small to large groups of people would find the TC environment challenging. Although the previous studies did not examine psychiatric comorbidities, it appears that social anxieties might be problematic for individuals remaining in residential treatment due to the highly social nature of the TCs. There is substantial evidence to suggest that social anxiety and substance use problems frequently co-exist, and that substance users with co-occurring social anxiety are significantly more impaired than substance users with no substantial social anxieties see [24]. Thus, it is likely that social anxieties may impede actual entry into residential treatment for substance dependence. That is, unlike depressive symptoms which can sometimes increase the likelihood that an individual seeks substance misuse treatment [25], it is possible that social anxiety may serve as a barrier to seeking treatment for substance misuse problems, particularly group or residential treatment. This is not surprising given the highly social nature of these two treatment modalities.

Social anxiety is pervasive and disabling and is characterised by intense, persistent fear and anxiety of situations involving social interaction, social evaluation and appraisal [26]. It has been argued that social anxiety exists on a continuum, with social anxiety at the mild end of the continuum, manifesting as shyness, and at the extreme end as the clinical diagnostic categories of Social Anxiety Disorder (SAD) in the DSM-5 (APA, 2013) and Social Phobia in the ICD-10 (WHO, 1992) [World Health Organisation [WHO], 1992; [27]. The DSM-5 defines SAD as a “marked fear or anxiety about one or more social situations in which the individual is exposed to possible scrutiny by others” (Criterion A; p. 202), with the individual fearing that they will act in a way or show anxiety symptoms that will lead to them being negatively evaluated (Criterion B). These social situations almost always provoke fear or anxiety (Criterion C), are avoided, or endured with intense fear or anxiety (Criterion D), with the fear or anxiety reaction being judged to be out of proportion with the actual threat posed by the social situation (Criterion E).

According to National Comorbidity Survey data [28,29], SAD is the most commonly reported anxiety disorder. In a nationally representative Australian population study, Teesson, Hall, Lynskey and Degenhardt [30] found that 6.5% of Australian adults met ICD-10 criteria for alcohol use disorder. Of these individuals, 3.7% met criteria for SAD, and among individuals who met criteria for SAD, 16.7% met criteria for an alcohol use disorder. Among adults seeking drug or alcohol treatment, rates of SAD are considerably higher, with comorbidity rates of up to 56% reported [31,32]. While the causal relationship remains unclear, concurrent social anxiety seems to lead to social isolation and depression in those recovering from substance dependency, which in turn increases the likelihood of drug or alcohol relapse and the reliance on drug use in social situations i.e., self-medication and tension-reduction; [33]. Thus, individuals with comorbid SAD are at greater risk of poor treatment retention [11,12] and relapse to substance misuse than those with substance misuse problems alone. Furthermore, although depressive symptoms can be ameliorated to some extent by treatment for substance misuse alone see [34], social anxiety disorder remains at clinical levels after drug treatment despite reductions in substance use [35] and has been shown to be risk factor for relapse [36].

Although most research and treatment models tend to categorize social anxiety as a categorical phenomenon, it has been argued e.g., see [37] that social anxiety is better represented dimensionally. Preliminary evidence supporting a dimensional conceptualization of social anxiety came from studies which attempted to form subgroups based on symptom severity. For example, although Stein, Torgrud and Walker [38] were able to classify individuals into three groups based on the number of social situations they feared (e.g., 1 to 3 social fears, 4 to 6 social fears and 7 to 12 social fears) they found that disability was related to the number of situations feared in a continuous manner, which indicated that viewing the number of social fears dimensionally was potentially more appropriate than viewing them categorically. Similarly, Vriends, Becker, Meyer, Michael and Margraf [39] found that when socially anxious individuals were classified into groups according to symptom severity, that there was no clear boundary between the subtypes.

More conclusive evidence for the dimensionality of social anxiety has come from taxometric analyses. Using data from the National Comorbidity Survey Replication (NCS-R), Ruscio [40] conducted a number of taxometric analyses (MAMBAC, MAXEIG and L-Mode) and found strong support for a dimensional, rather than taxonic structure. These results were replicated by Crome, Baillie, Slade and Ruscio [41] whose analyses of both the NCS-R dataset and the Australian National Survey of Mental Health (ANSMH) dataset also strongly supported the dimensional nature of social anxiety. Importantly, support for the dimensional nature of social anxiety has also been found in clinical populations, with Kollman, Brown, Liverant and Hofmann [42] finding support for the dimensional nature of social anxiety in a sample of 2,035 outpatients presenting for treatment at a US based anxiety disorder treatment centre.

From a clinical perspective, the usefulness of considering social anxiety dimensionally was demonstrated by Ruscio [40] who found that dimensional SAD was a better predictor than categorical SAD of a number of outcomes, including subsequent suicide ideation, mood disorders, and treatment seeking behaviours. From the perspective of alcohol use, Crum and Pratt [43] found that individuals with subclinical social anxiety symptoms were more likely to have drinking problems than those with a clinical diagnosis of SAD, indicating consideration of social anxiety as a dimensional, rather than categorical phenomenon might be especially important in research exploring social anxiety and alcohol and substance use disorders.

Taken together, the results of these studies is that differences between shyness, social anxiety, and avoidant personality disorder are quantitative, rather than qualitative (although for an alternative position, see [44]). These results have implications for clinical research on social anxiety and suggest that an exclusive focus on individuals who meet DSM criteria for SAD is not clinically warranted. Moreover, as Kollman, Brown, Liverant and Hofmann [42] note, it also suggests that social anxiety ought to be assessed using continuously, rather than forced choice measurements.

Currently, social anxiety in individuals seeking treatment for substance misuse social anxiety is poorly detected [45] and inadequately treated within alcohol and drug treatment settings [46]. Indeed, very few studies have attempted to address social anxiety in individuals presenting for substance use disorders [33]. This highlights the possible need for targeted treatment for individuals with social anxiety and comorbid substance misuse. Given that social anxiety may play a role in an individual deciding not to commence or continue residential treatment, it is argued e.g., [47] that addressing social anxiety symptoms prior to and/or at the early stages of treatment could improve treatment retention in individuals with comorbid social anxiety and substance misuse problems. Considering the complex nature of the relationship between SAD and substance misuse, it is unclear how to proceed when treating SAD in individuals with substance misuse problems. Specifically, it is unclear if SAD should be treated first or concurrently with treatment for substance misuse problems. Currently, only two RTCs have explored the efficacy of treating SAD in substance use populations [5,48].

The first study [5] involved individuals seeking treatment for alcohol use problems who met criteria for both alcohol misuse and SAD. Individuals were randomised into one of two treatment conditions, receiving either a 12 week individual cognitive behavioural therapy (CBT) intervention targeting the alcohol problem only or a 12 week individual CBT treatment targeting both alcohol misuse and social anxiety problems (which they referred to as the dual group). In the alcohol only intervention group, treatment sessions lasted 60 minutes. In the dual group, both alcohol and social anxiety was covered in each session (beginning with alcohol and ending with social anxiety). Unexpectedly, there were no differences between the alcohol only and dual intervention groups on social anxiety symptom severity. Furthermore, the dual intervention group reported poorer alcohol-related outcomes compared to the alcohol only intervention group. The authors noted that a limitation of their methodology was the somewhat segregated approach to treating both disorders. Specifically, the concurrent sessions were divided into two sections during the one treatment session: treatment of the alcohol problem, followed by CBT for social anxiety. The effectiveness of this temporal within-session sequence was questioned by the authors and it was noted that the literature is unclear on the best approach.

A second study [48] compared the efficacy of an intensive psychosocial relapse-prevention program delivered on its own or in combination with an anxiety treatment program comprising CBT and optional pharmacotherapy (i.e., selective serotonin re-uptake inhibitors; SSRI). Although the addition of the anxiety treatment program did result in significant reductions to anxiety symptoms and avoidance, the program was not associated with concomitant reductions in alcohol relapse rates. Despite this, there was a non-significant trend for the combined treatment to be associated with a greater likelihood of abstinence of at least 30 days relative to the alcohol only intervention group, with an effect size of 0.13. It has recently been noted, however that the small sample size might have meant that the study was underpowered [49].

Taken together, there is some evidence to suggest that treatment of social anxiety in treatment seeking substance users results in reductions in anxiety symptoms, however, the effects on substance misuse are less clear. Currently, there are no studies which examine whether addressing social anxiety prior to treatment improves rate of treatment entry and subsequent treatment retention. This is important, because as noted earlier, length of tenure in TC treatment is a significant predictor of improved treatment outcomes. Thus, the focus of the current study is on examining whether addressing social anxiety symptoms prior to entry for residential treatment improves the likelihood that individuals will enter and stay in treatment. A secondary goal, in line with previous clinical studies, is to explore if treatment of social anxiety results in concomitant reductions in other indices of psychological distress (e.g., depression and general levels of anxiety).

The current study utilises a randomised control design which adheres to CONSORT guidelines [50]. The intervention group receives four sessions of treatment for social anxiety symptoms, plus boosters, and the control group remain on the waiting list for entry into residential treatment (as a treatment as usual group). The proposed treatment program for SAD is based on Rapee’s [51] program “Overcoming Shyness and Social Phobia: A Step by Step Guide”. This treatment approach involves a range of standard empirically validated methods for addressing social anxiety disorder symptoms, including: attention training, cognitive restructuring, exposure to feared situations and realistic feedback of social performance. Rapee’s [51] program focuses on setting ‘homework’ for clients, primarily on restructuring beliefs about social situations into more realistic terms. It is designed to be undertaken as either ‘pure self-help’ or ‘therapist augmented self-help’ where clinicians help ‘problem-solve’ the program’s concepts with a specific focus on each client’s personal context [52] Two RCTs support the effectiveness of the program in reducing social anxiety symptoms and other related psychological problems [52,53].

The current study sought to examine whether treatment of social anxiety prior to entry to treatment would increase the likelihood that individuals would enter, and subsequently remain in treatment for at least three months. Additionally, the current study focusses on whether brief treatment of social anxiety would result in significant reductions in social anxiety symptom severity over time, and significant reduction in other indices of psychological distress.

## Research objectives

### Primary hypotheses

1. It is hypothesised that individuals in the intervention group will be more likely to enter the TC than individuals in the control group.

2. It is hypothesised that individuals in the intervention group will be more likely than individuals in the control group to stay in treatment for at least three months.

### Secondary hypotheses

1. It is hypothesised that participants in the intervention group will report significant reductions in social anxiety severity between baseline (T1) and post-intervention (T2) assessments.

2. It is hypothesised that among participants who met Mini criteria for SAD at baseline (T1), that significantly more participants in the intervention group will no longer meet criteria for SAD at the three month follow-up (T3) relative to the control group.

3. It is hypothesised that participants in the intervention group will report significantly fewer symptoms of anxiety and depression following treatment of social anxiety than participants in the control group.

## Method/design

### Study design

The study design is a randomised control trial where the intervention is being compared to treatment as usual. Enrolment is conducted at Odyssey House Victoria (OHV), an addiction treatment facility located in Melbourne, Australia. Enrolment commenced June 2010 and is ongoing. All procedures are conducted in accordance with the Australian Code for the Responsible Conduct of Research and were approved by the Deakin University Human Research Ethics Committee.

### Study participants and treatment setting

Participants are individuals who apply for entry to a therapeutic community (TC) based residential rehabilitation treatment program provided through OHV for alcohol and substance use problems.

### Procedure

**Figure 1:**
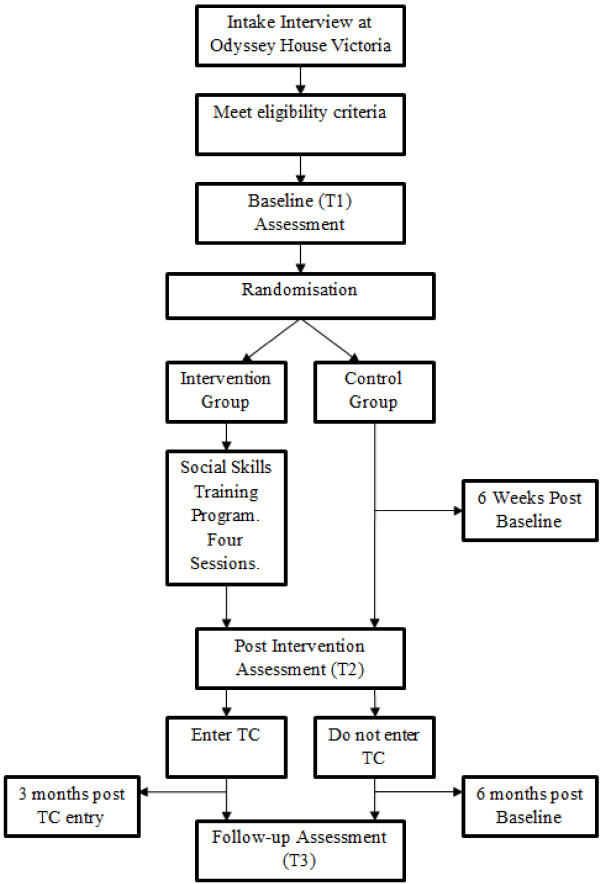
Study procedure flowchart.

The following sections describe the Study procedure. See Figure 1 for an overview.

#### Initial screening

Intake assessments are conducted at OHV in order to determine client suitability for treatment. As part of the standard intake assessment clients complete the self-report version of the Liebowitz Social Anxiety Scale – Self-Report version (LSAS [54]). If the client meets eligibility for the study the intake team provides them with information about the study and offers that an independent research team member could contact them to provide information on the study. Given the self-report format employed, only clients who attended intake assessments in person were given an LSAS to complete, and the LSAS was not offered in the case of phone interviews. The LSAS was offered to clients who are new to OHV, as well as those who had previously been residents but who are seeking re-entry into the program due to relapse.

#### Inclusion criteria

1. Participants need to be deemed eligible for entry into the TC by OHV clinicians.

2. Participants must meet criteria for substance dependence or alcohol dependence as defined by the DSM-IV-TR [55].

3. On the basis of the strong support for the dimensional nature of social anxiety (e.g., [41,42]) and on evidence suggesting that individuals with sub threshold social anxiety are particularly vulnerable to substance use problems , the decision was made to base study inclusion on a dimensional scale, rather than on a diagnosis of SAD. For inclusion in the study, participants must report sub clinical levels of social anxiety as measured by the LSAS. Specifically, they must report at least one moderate symptom of social anxiety and report avoidance of at least one situation. This is consistent with Merikangas, Avenevoli, Acharyya, Zhang, and Angst [56] who indicated that this level of symptomatology represented sub clinical levels of social anxiety.

#### Exclusion criteria

1. Participants are excluded in they are under the age of 18 years

2. Participants are excluded if there is evidence of florid or active psychosis

3. Participants are excluded if they report current, severe suicidality

4. Participants are excluded if they are unable to read English.

Participants who meet the inclusion criteria were invited to participate in the study. Participants were advised that their participation is optional and would not affect their eligibility or wait time for the rehabilitation program.

#### Baseline assessment (T1)

Baseline assessments are administered using a standardised protocol. All researchers received prior training on the instruments used in the assessment prior to conducting interviews. In addition, new research officers conducted a number of practice assessments with a clinical psychologist at OHV prior to working with clients. The first assessment conducted by new research officers was also co-administered by a clinical psychologist at OHV, in order to ensure adherence to the assessment protocol, and to assess interrater agreement. Research officers are fully briefed as to the requirements associated with duty of care, and are made aware of the safety protocols outlined by OHV. Given the time commitment associated with the baseline (approximately 1 ½ - 2 hours) participants are reimbursed with a $25 retail voucher. For a list of measures used in the baseline assessment, see Table 1.

**Table 1:** Measures administered at each assessment

**Measure**	**Baseline (T1)**	**Post intervention (T2)**	**Follow-up (T3)**
Demographic questions	✓	-	✓
TLFB	✓	-	✓
MINI	✓	-	✓^a^
ASSIST	✓	-	✓
LSAS	✓	✓	✓
BAI	✓	✓	✓
BDI	✓	✓	✓
CMR	✓	✓	-
CSQ-8	-	✓^b^	-

*Note.* TLFB = Timeline Follow-back Method, MINI = Mini International Neuropsychiatric Interview, ASSIST = Alcohol, Smoking and Substances Involvement Screening Test, LSAS = Liebowitz Social Anxiety Scale, BAI = Beck Anxiety Inventory, BDI = Beck Depression Inventory-II, CMR = Circumstances, Motivation and Readiness for Treatment Scale, CSQ-8 = Client Satisfaction Questionnaire-8.

^a^only modules F, I and J are administered. ^b^only administered to intervention clients.

#### Randomisation

Following the baseline interview, an independent researcher randomises participants to either the intervention or control group. A permuted block randomisation procedure is utilised (e.g., Altman et al., 2001) whereby participants are allocated to the intervention or control group through the use of a randomly generated number. The permuted blocks are organised in groups of four (e.g., AABB, ABAB, BBAA) the details of which are not known by investigators involved with the administration of the trial. A random number sequence is generated which indicates the block of four conditions to be utilised for the following four cases. The use of the permuted block randomisation process ensures that intervention group numbers are balanced at the end of each block and is thus the recommended process in studies with smaller samples (i.e., n < 100).

#### Intervention group

Participants in the intervention group receive the Social Skills Training Program adapted from Rapee’s training program. The program was developed in consultation with Professor Rapee. The program consists of two individual and two group sessions held at OHV. Group sessions include a maximum of four participants. When there are insufficient currently enrolled participants to form a group, the final two sessions are conducted as individual sessions. All sessions are approximately one hour long. One month after the final intervention session, if clients have not entered the TC, a booster session is run, which involves a brief 15 minute review of the content of the four sessions, and is conducted by the clinical psychologist. If the client has not entered the TC a month after the first booster session, a second booster session is conducted. Once clients have entered the TC, two booster sessions are run within the first month of a client’s tenure at the TC. Booster sessions at the TC follow the same format as those conducted prior to entry to the TC.

The sessions are facilitated by a registered and experienced psychologist and conducted according to a structured manual that includes handouts and information sheets. Sessions are conducted while the participant is on the wait list for entry into the TC. The average time between sessions was one week.

The program material was developed using principles of cognitive behaviour therapy and was guided by the work of Rapee (1998). The program content included; social anxiety psychoeducation, cognitive challenging, reality testing and attention training (Rapee, 1998). In addition, planning for challenges/setbacks that may arise prior to or upon entry into the TC is a facet of the later sessions. Each session is structured to optimise coverage of the topic material and a specific checklist of session goals is used by the clinician during each phase of the intervention. At the end of each session the client completes a measure assessing their ability to concentrate as well as their attitudes towards the session content. Participants are also asked to complete homework assignments which are reviewed at the beginning of each session. Table 2 outlines the content covered in the four sessions.

**Table 2:** O**verview of social skills training program**

**Session**	**Content**
Session 1: psychoeducation	• Introduction
	• Provide therapy rationale anxiety and social anxiety psychoeducation
	• Introduce and personalise model of social anxiety
Session 2: cognitive model	• Review model of social anxiety & treatment rationale
	• Introduce importance of realistic/helpful thinking
	• Generate unhelpful/unrealistic thoughts with client
	• Introduce more realistic/helpful thinking
Session 3: attention and behavioural experiments	• Group introduction and guidelines
	• Review rationale and goals for social anxiety group
	• Review cognitive component and model of social anxiety
	• Introduce attention training/breathing exercise
	• Conduct attention behavioural experiments
Session 4: review and relapse prevention	• Review week and homework
	• Review social anxiety model
	• Review treatment components
	• Discuss nature of recovery and relate to upcoming challenges
	• Relapse prevention planning
	• Administer post-intervention questionnaires

#### Therapist adherence

All therapists underwent training in the administration of the Social Skills Training Program. All sessions are recorded in order to monitor content and competency. Participants provided verbal consent to be recorded and are not identified by name during the session. The therapists have regular meetings with a registered clinical psychologist with recognised clinical supervision qualifications and expertise in the cognitive-behavioural treatment of social anxiety. At the conclusion of the study, a random sample (10%) of recorded interviews will be reviewed to monitor content and competency.

#### Control group

Participants in the control group receive an information sheet restating the standard protocol of admission procedures to the TC and encouraging them to attend the preparation sessions which is standard for all participants waiting to enter. Participants in the control group also receive a brief information on the definitions of anxiety (e.g., symptoms, prevalence). The time between the administration of the initial baseline assessment and admission into the TC varied.

#### Post intervention assessment (T2)

At the conclusion of the fourth session participants in the intervention group complete a post intervention assessment. Participants in the control group complete the same assessment six weeks after their baseline assessment. In this post intervention assessment, participants answer qualitative questions relating to the information received in each condition. In the event that a participant enters the TC prior to completion of the post intervention measure, assessment is conducted at the TC. For a list of measures used in the T2 assessment, see Table 1.

#### Follow up assessment (T3)

Three months after entering the TC, all intervention and control group participants are asked to complete a follow up interview, which includes qualitative questions relating to their time spent at the TC. In instances where a client does not enter the TC, the T3 is administered six months after the baseline assessment. For a list of measures used in the T3 assessment, see Table 1.

### Measures

Assessment information for this study was drawn from semi-structured clinical interviews and self-report questionnaires. In addition clinicians trained in mental health assessment administered standardised questionnaires and clinical assessment tools. A list of measures used at each assessment point is provided in Table 1.

### Clinician administered measures

#### Demographic questionnaire

A basic demographic questionnaire was used to capture client characteristics and included questions relating to age, education, socio-economic status as well as personal and familial history of substance use and mental health issues.

#### Mini International Neuropsychiatric Interview

The MINI [57] is a short, structured diagnostic interview, used to diagnose depression, anxiety, psychotic and substance use disorders from DSM-IV criteria. It takes approximately 15 minutes to administer and uses a decision logic tree to assess the major Axis I disorders in the DSM-IV-TR and ICD-10. The MINI is reported to demonstrate high reliability and good concordance with other diagnostic measures such as the Composite International Diagnostic Interview and Structured Clinical Interview DSM-III-R-Patients [57,58]. The MINI was administered at T1 to clinically assess all potential diagnoses identified from the MINI-Screen. All participants are administered the sections assessing social anxiety disorder, antisocial personality disorder, alcohol use and substance use disorder. The social anxiety, alcohol use and substance use disorder sections of the MINI are also administered at T3.

#### Alcohol, Smoking and Substances Involvement Screening Test (ASSIST)

The ASSIST [59] is a reliable and valid screening test for problematic or risky substance use. Eight questions assess cannabis, cocaine, amphetamines, inhalants, sedatives, hallucinogens, opiates and other miscellaneous drugs, alcohol and tobacco use within the context of a more general health and lifestyle-screening interview. Australian research indicates that it is a valid screening test for substance use in individuals who use a number of substances and have varying degrees of substance use [60].

#### Timeline Follow-Back method (TLFB)

The TLFB [61] measure was used to determine quantity and frequency of alcohol and drug use. The TLFB is an established calendar-based assessment tool created to assist client recall of substance consumption over a desired time period using date-based memory triggers. Use in the previous 90 days was recorded.

### Self-report measures

#### Liebowitz Social Anxiety Scale (LSAS)

The LSAS [54] is a 24 item scale assessing levels of fear and avoidance across a range of social and performance interaction situations. Items are rated on a four point scale, with higher scores indicative of more severe social anxiety. SAD is determined by an empirically-derived cut-off score e.g., [29,62]. The LSAS has been found to possess acceptable psychometric properties [63], and has been found to be sensitive to changes in social anxiety following treatment. Although originally used as a clinician administered scale, research has shown the self-report version to be equivalent [64].

#### Beck Anxiety Inventory (BAI)

The BAI [65] is a 21- item measure of the severity of anxiety in psychiatric populations. Participants rate the severity of each symptom of anxiety on a 4-point Likert scale (0 = not at all, to 3 = severely - I could barely stand it). Items are summed for a total score. The BAI demonstrates good internal consistency, test-retest reliability and convergent validity with Hamilton Anxiety Rating Scale–Revised (Beck et al., [65]).

#### Beck Depression Inventory-II (BDI)

The BDI [66] consists of 21 items measuring common symptoms of depression on a 4-point Likert scale (0 = not at all, to 3 = severely - I could barely stand it). Items are summed for a total score. The BDI demonstrates high internal consistency, test-retest reliability and convergent validity with the Hamilton Rating Scale for Depression–Revised [67]

#### Circumstances, Motivation and Readiness for Treatment Scale (CMRS)

The CMRS is a 20 item scale [68] developed as a self-report scale of client perceptions across four interrelated domains: circumstances (external pressure), motivation (intrinsic pressure), readiness and suitability for residential treatment. Scale items are rated on a 5-point likert scale from 1 (Strongly Disagree) to 5 (Strongly Agree) with the option to select N/A if the question does not apply to the respondent. The CMRS was found to have acceptable internal consistency (.87 for the total scale), and was found to be predictive of treatment durations of longer than 30 days.

#### Client Satisfaction Questionnaire-8 (CSQ-8)

The CSQ-8 [69] is an 8-item questionnaire designed to measure client satisfaction with a particular health-care service, and was used to evaluate how satisfied the clients were with the social anxiety intervention. The CSQ-8 has good internal reliability, ranging from .83 to .93; while high construct validity has also been demonstrated [69]. The CSQ-8 has been translated into 15 different languages, and is utilised across a wide range of populations and health services [69].

### Qualitative questions

In addition to the structured measures, qualitative questions developed specifically for the study are asked at T2 and T3. These questions focused on the expectations of the participant and their opinion of the intervention program and treatment program at the TC.

## Data analysis

1. Differences between the intervention and control groups in categorical DVs (entry to the TC, TC stays of at least 30 days, 45 days and 90 days) will be analysed using binary logistic regression chi-square analysis.

2. Differences between the intervention and control groups in time spent at the TC will be analysed using Analysis of Variance, and Cox Proportionate Hazard Models.

3. Differences between the intervention and control groups in psychological outcomes over time (i.e., LSAS, BDI and BAI) will be explored using linear mixed modelling. In order to select the appropriate correlation structure for each of these DVs, linear models will initially be fitted using one of three possible correlation structures; compound symmetry (non-zero uniform correlations and uniform variance between time points), first order auto-regressive (observations closers in time are more highly correlated than observations further apart), and scaled identity (uniform variance across time with zero correlations between time points). The best fitting model for each outcome variable will be decided by selecting top fitting models based on Akaike’s Information Criterion (AIC).

4. Differences between the intervention and control groups in number of participants who no longer meet Mini criteria for SAD at the three month follow-up will be assessed using a 2 (intervention vs. control group) × 2 (SAD vs. no SAD) chi-square analysis. This analysis will only be conducted with participants who met Mini criteria for SAD at the baseline assessment (T1).

### Power analysis

Power analysis indicates that an overall sample size of 121 is required to detect a medium effect size (approx .70) at the .05 alpha level using linear techniques (power = .80). On the basis of a previous study conducted at Odyssey House Victoria [70] it is expected that approximately 21% will be lost to follow up hence the target of the current study will be a sample of 146.

## Discussion

Individuals presenting for residential drug rehabilitation programs report substantially worse psychiatric comorbidities than outpatients. A large proportion of those claiming to seek treatment will not enter treatment programs, and of those who do enter treatment, rates of premature dropout within the first five weeks of treatment are high. This is particularly problematic given the body of evidence suggesting that for residential treatment modalities treatment tenure of at least three months is associated with substantially better treatment outcomes. Indeed, some studies have indicated e.g., [71] that it is not until after the three month period that there emerges a correlation between time spent in treatment and treatment outcomes. Substantial literature has reported that social anxieties frequently co-occur with substance use problems, and that individuals with comorbid social anxiety and substance use problems have a poorer prognosis than those with no substantial social anxieties. There is some preliminary evidence that number, and quality of social contacts prior to substance use is a significant predictor of treatment retention. In addition, a number of clinical trials have explored the effect of treating social anxiety on treatment outcomes, and although there is evidence that such treatment results in improvements to anxiety symptoms, this was not related to concomitant reductions in substance use severity. One of the limitations of these studies, however, is that they focussed on examining whether treatment of social anxiety was related to reductions in social anxiety severity and improvements in substance use problems, but did not explore the effect of treatment of social anxiety on entry into treatment and on treatment retention. Given the substantial literature supporting the efficacy of TCs in cases where treatment exceeds the three month threshold, the current study sought to explore whether treatment of social anxiety prior to entry into treatment will increase the likelihood of entry into residential treatment and decrease the likelihood of premature treatment dropout. The results of the study will have implications for addressing social anxiety within residential drug treatment services. The results might suggest that the use of additional screening tools in intake assessments, a focus on coping with social anxieties in support groups for clients waiting to enter treatment, and greater awareness of social anxiety issues among residential rehabilitation staff is warranted.

## Competing interests

The authors declare that they have no competing interests.

## Authors’ contributions

PS. Design of the study, development of the intervention, manuscript preparation, final approval of manuscript. MK. Design of the study, development of the intervention. JW. Manuscript preparation, data collection. NK. Design of the study, statistical design. AH. Development of the intervention. SG. Design of the study, development of the intervention. All authors read and approved the final manuscript.

## Pre-publication history

The pre-publication history for this paper can be accessed here:

http://www.biomedcentral.com/1471-244X/14/43/prepub
